# Finite Element Analysis of Plastic Deformation in Ultrasonic Vibration Superimposed Face Milling of Steel X46Cr13

**DOI:** 10.3390/mi15060730

**Published:** 2024-05-30

**Authors:** Richard Börner, Philipp Steinert, Nithin Kumar Bandaru, Andreas Schubert

**Affiliations:** Professorship Micromanufacturing Technology, Chemnitz University of Technology, Reichenhainer Str. 70, 09126 Chemnitz, Germany; philipp.steinert@mb.tu-chemnitz.de (P.S.); nithin-kumar.bandaru@mb.tu-chemnitz.de (N.K.B.)

**Keywords:** Abaqus, numerical simulation, microstructure, ultrasonic vibration superimposed machining, X46Cr13

## Abstract

Ultrasonic vibration superimposed face milling enables the generation of predefined surface microstructures by an appropriate setting of the process parameters. The geometrical reproducibility of the surface characteristics depends strongly on the plastic material deformation. Thus, the precise prediction of the emerging surface microstructures using kinematic simulation models is limited, because they ignore the influence of material flow. Consequently, the effects of plastic as well as elastic deformation are investigated in depth by finite element analysis. Microstructured surfaces resulting from these numerical models are characterized quantitatively by areal surface parameters and compared to those from a kinematical simulation and a real machined surface. A high degree of conformity between the values of the simulated surfaces and the measured values is achieved, particularly with regard to material distribution. Deficits in predictability exist primarily due to deviations in plastic deformation. Future research can address this, either by implementing a temperature consideration or adapting specific modeling aspects like an adjusted depth of cut or experimental validated material parameters.

## 1. Introduction

Ultrasonic vibration superimposed machining (UVSM) offers the possibility of generating predefined surface microstructures by setting various coordinated process parameters. These microstructures obtain the possibility of creating functional properties, such as promoting static friction, reducing wetting behavior or improving layer adhesion [1,2]. Their reproducibility depends to a large extent on their actual characteristics compared to the desired or idealized surface microstructure. In particular, it is assumed that the plastic, material-dependent deformation in the course of mechanical loading has a significant influence and leads to geometric deviations compared to an only kinematically simulated microstructure. In this paper, these effects are investigated in more detail by means of finite element (FE) analysis. Therefore, the example of the microstructuring of the martensitic stainless steel X46Cr13 by means of ultrasonic vibration superimposed face milling is chosen, in order to be able to use this kind of simulation for structural design if necessary.

Due to the complexity between the tool motion and resulting surface microstructure, the analysis of UVSM by numerical simulation can support the investigation of the formation principles of the shape of the surface microstructure. Modeling approaches to simulate the superimposed process can be divided into two categories: kinematic simulations and FE simulations. While the kinematic simulations mainly investigate the resulting surface microstructure, numerical approaches tend to focus on the design of process components/tools, thermal effects, chip formation and the prediction of cutting forces, respectively.

In some cases, purely analytical models are sufficient to predict the geometric properties of surface microstructures. This has been shown by Jerez-Mesa on the basis of calculations and experimental investigations of radial ultrasonic vibration-assisted machining on C45 [3]. In addition, for predicting the resulting surface microstructure in cutting processes, kinematic simulations are also relevant and suitable. These are mostly carried out using special and temporal discretization methods, as presented by Arizmendi and Jiménez [4] for face milling operations. In the common way, MATLAB software is used, as shown, e.g., by Freiburg and Biermann [5] for a similar process. Furthermore, relatively complex kinematic–geometric relationships, such as a vibration superposition, can be easily represented and varied independently of the material. Since comparative experimental implementations often take place on materials with good machinability (such as aluminum alloys) and with very sharp cutting edges (mostly polycrystalline or natural diamond), the significance of effects such as burr formation or elastic–plastic deformation, which cannot be represented with this simulation method, decreases.

For example, Lu et al. and Chen et al. used kinematic MATLAB simulation models to evaluate the agreement between predicted and experimentally generated surface microstructures. The investigations focused on milling processes that were superimposed once [6] or twice [7] with vibrations in the ultrasonic range in different spatial directions, but parallel to the machined surface.

However, there are also a few research studies in which the generation mechanism for the surface microstructures results from a superimposed oscillation perpendicular to the machined surface, which complies with the direction of the passive force. For ultrasonic vibration superimposed machining, the kinematics in turning and milling are very similar, regardless of whether the tool or workpiece is excited by vibration. However, the geometric properties of the microstructures produced differ depending on the process parameters and tool specification. While it is still possible to achieve different geometric characteristics of microstructures in vibration superimposed processes in the three-digit-to-low-four-digit Hz range, as investigated by Yuan et al. [8] and Zhu et al. [9], processes in the ultrasonic range are already much more difficult to predict. In particular, the displacement of the microstructures per subsequent feed path can only be controlled to a limited extent, as found, e.g., in [10,11], respectively. Liu et al. [10] had this experience with ultrasonic vibration superimposed turning of copper 1100. In their study, a combined approach, utilizing both theoretical models and experimental investigations, was employed to analyze the impact of tool geometry on the generated microstructures. It was also found that due to their superior sharpness and reduced wear, diamond cutting tools were preferred to cemented carbide tools. Despite the excellent machining conditions, structural deviations in comparison to the simulation were found, particularly in the overlapping parts, which indicates plastic deformation, among other things. An example for this is shown in Table 1. Since microstructuring was implemented in a turning process, there are no problems with recutting effects. However, this is a challenge in vibration superimposed milling operations. Therefore, Zheng et al. [11] developed so-called oblique rotary ultrasonic milling. To avoid the recutting effect, the tool was tilted in the direction of feed motion. In these studies, kinematical and numerical simulations were used and compared to the results of real microstructured surfaces, based on cutting experiments on aluminum and titanium alloys with an oscillating tool.

To compare some representative microstructures regarding their shape and dimensions, which are similar to the mentioned research topic of this paper, some examples from the literature are depicted in Table 1.

A model for the kinematic simulation of the surface microstructure for UVSM processes was also developed at our department. In the simulation function, the workpiece Dexel is transformed into the tool coordinate system by moving and rotating it around the *Z*-axis (the rotational axis of the tool) and then comparing it with the nearest tool Dexel. By gradually applying smaller height values along the path vector, the individual cutting behavior of the tool is guided to an approximately continuous cut and a high process resolution is ensured [12]. The resolution has a significant impact on the efficiency of the simulation. The spatial resolution of the workpiece and the tool is fixed and proportional to the simulation time and the imaging accuracy of the surface microstructure, while the temporal resolution depends on the process parameters. Therefore, the simulation model can automatically determine the minimum required time resolution based on the number of cuts per period and frequencies in the ultrasonic range [13].

However, it is clear from the illustrations and results that there are minor-to-major differences between kinematically simulated microstructures and real generated surfaces for each machining scenario. Depending in particular on the material to be machined and the tool used (wear condition, cutting material), but also on when external influences (friction, e.g., reduction due to lubrication, temperature changes) are (not) taken into account, simulated and real microstructures sometimes deviate significantly from each other. In critical cases, simulation methods that can incorporate other, particularly plastic effects, could therefore be advantageous.

Research on the surface generating mechanism of vibration superimposed cutting supported by numerical simulations is relatively poorly documented compared to that of kinematic simulations. As mentioned above, FE models are mostly related to cutting forces, chip formation or thermal conditions. Only a few papers deal with numerical simulations in the context of surface microstructures.

Korkmaz et al. and [14] Elkaseer et al. [15] simulated the turning of steel AISI 420 (equated to X46Cr13) and a similar alloy (316L) by applying cemented carbide tools. The FE analyses used, among other things, the Johnson–Cook (JC) elastic–plastic material model and the JC damage model as well. This is discussed in more detail below. The main focus of the investigations was on the prediction of the forces [14] and the chip formation [15].

A comprehensive literature review study was conducted by Lotfi and Akbari [16]. Different types of vibrational conditions, employed in ultrasonic vibration-assisted machining simulation, were explored, including linear, elliptical and longitudinal-torsional vibrations. The authors identified several key areas that require further attention to enhance simulations for vibration-assisted machining processes, especially in the ultrasonic range. One of these topics is the consideration of microstructures generated during elliptical vibration, which are often disregarded during simulation due to the remeshing process of these small microstructures. Additionally, the authors emphasized the need for specialized simulation models (flow stress, tool wear, etc.) developed for ultrasonic superimposed processes, rather than relying on conventional machining models, as the high-frequency oscillation can significantly impact the model parameters and simulation results.

As previously mentioned in the studies by Zheng et al. [11] on oblique rotatory ultrasonic milling, in addition to the kinematic simulation of the resulting microstructure, an FE model was also developed to simulate the burr formation, but also temperature changes and resulting residual stresses, using the example of a single cut. For this purpose, a simplified linear movement in the direction of the cutting speed was assumed and supplemented by the effective tool vibration as a resulting sinusoidal curve. The JC models for deformation and damage were also used.

Mitrofanov et al. [17] demonstrated that ultrasonic vibration-assisted turning can significantly reduce cutting forces, heat and noise while improving surface finish compared to conventional machining of Inconel 718. Their research involved applying high-frequency vibration (20 kHz) with an amplitude (up to 10 µm) to the cutting tool movement in the cutting direction. A suggested FE model offers transient analysis for elastic–plastic material behavior. It considers frictionless contact between the cutting tool and the workpiece and material separation ahead of the cutting edge.

Abootorabi Zarchi et al. [18] discussed the application of ultrasonic vibration on side milling in the direction of feed motion on AISI 420 stainless steel. They presented a new model for predicting cutting forces and examining the effects on surface roughness values and cutting forces. The study revealed that this kind of added movement can lead to lower cutting forces and improves surface roughness under certain conditions, particularly with low feeds and high cutting speeds during up milling. The research indicates that the process has the potential to enhance the milling process of materials such as AISI 420 stainless steel, which is commonly used in high-strength applications.

Ying et al. [19] introduced longitudinal-torsion ultrasonic vibration-assisted milling as a method to enhance the machining of difficult-to-cut materials, such as titanium alloys, with a particular focus on improving the fatigue strength by manufacturing. Using 3D finite element simulation, analyses of cutting force, cutting temperature and residual stresses were conducted. A thermal-mechanical coupled numerical simulation model of ultrasonic vibration-assisted milling was established, followed by the development of an equivalent 3D FE model for the longitudinal-torsional movement to improve computation efficiency. The results demonstrated that this machining process effectively reduced cutting force and temperature while increasing the absolute values of the surface residual compressive stresses. Experimental verification agreed with simulation outcomes, confirming the precision of the model in predicting suitable machining parameters. The study demonstrates the efficiency of longitudinal-torsion ultrasonic vibration-assisted milling in enhancing the machining of titanium alloys, providing insights into its potential for increasing the fatigue strength by manufacturing.

Previous work in our department in the area of FE simulation of machining was conducted to establish a novel process for the fabrication of microstructures that can be used to realize improved bond strength for a metal–polymer interface. Ultrasonic vibration-assisted deformational machining was investigated to generate these microstructures at high production rates. An FE simulation by the software Abaqus was used to understand the burr formation during the machining of the aluminum alloy EN AW-6082 in the heat treatment condition T6. Various process parameters were considered in this simulation, including tool geometry and plastic material deformation described by the JC model. Experimental validation of the simulation results was performed with two different machining tools. During the tests, constant parameters were maintained: a fixed ultrasonic frequency of 20.42 kHz, machining speeds of 100 m/min and 200 m/min and a peak-to-peak amplitude of 7 μm. The geometric surface characteristics were analyzed using photo-optical methods and scanning electron microscopy (SEM). The results of the study show a close agreement between simulated and experimental results, underscoring the significant potential of this process to improve productivity in the surface modification of the metallic part for the generation of polymer/metal hybrid structures [20].

In most cases, FE models of cutting processes with additional movement consider chip formation, as well as stress and temperature distribution in the cutting zone, machining forces and the resulting loads on the tool or residual stresses in the surface boundary zone. Surface microstructuring, on the other hand, is rarely the subject of such analysis, as in many cases the resulting geometric surface information determined with the help of kinematic simulation methods is sufficient. Particularly with brittle materials or very sharp tool cutting edges, e.g., made of monocrystalline diamond, these can also be sufficiently accurate, as hardly any plastic deformation occurs. However, as the strength and toughness of the material to be machined and the cutting edge rounding increases, e.g., from a manufacturing point of view or due to tool wear, the predictability accuracy of the resulting microstructures of the UVSM process decreases. The development of an FE model considering the plasticity can come much closer to reality here. This could be used, for example, to design microstructured functional surfaces that increase layer adhesion or are tribologically effective for applications in various industrial sectors, such as aerospace, medical technology, forming technology or a variety of mechanical components.

## 2. Materials and Methods

The aim of these research studies was to identify the “deformative part” in relation to microstructure characteristics and accordingly surface parameters. To compare the kinematic simulation approach with a numerical simulation based on FE analysis and a real microstructured surface, the following steps were conducted: process parameters, geometrical specifications and tool properties were chosen from a UVSM reference process, in which the tool wear was very small. By using the 3D data of the tool without considering any wear, an idealized shape was developed. This approach was employed for conducting both kinematic and numerical UVSM simulations. Furthermore, the condition of this tool after UVSM of one specimen (780 mm^2^ area) was used for a comparative FE model, which additionally takes the real structural displacements into account. This is defined by an offset value of the structural elements, for example, the peaks, in the cutting direction from one feed path to the next feed path.

For the numerical simulation, four distinct structural displacements were established. Otherwise, only three models were required for the kinematic model, since the structural displacements of X.25 and X.75 were mirror-inverted and share identical surface parameters. By means of these different combinations, the “deformative part” or the resulting deviations for the kinematic simulation could be determined. An FE model adapted to the geometric aspects was then compared with a real reference surface.

### 2.1. Data from Experimental Studies

A microstructured specimen from the experiments on CVD diamond coating of steel by Göltz et al. [21] was used as the reference surface. Here, a section was recorded nearly at the end of the machining process. With a tool diameter of 35 mm and an ultrasonic frequency of *f*_US_ = 19.7 kHz, a cutting speed of *v*_c_ = 30 m/min was required to achieve a structural distance in the cutting direction of 25 µm. By setting the feed to *f* = 25 µm, this determines the structural distance in the direction of feed motion. The ultrasonic superimposition oscillates in a direction perpendicular to the specimen surface with a set amplitude of *A*_US_ = 2.5 µm. For surface characterization, a specimen was chosen which reached the desired ultrasonic amplitude values with almost no deviation. The tool used was a special indexable insert made of coated ultrafine-grain cemented carbide, which is described in more detail in [22].

#### 2.1.1. Surface Characterization

**Figure 1:**
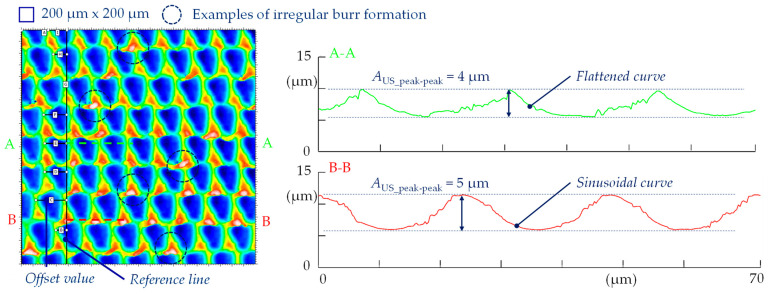
LSM-recorded reference surface section with measurements of the structural displacement from the top view and the real achieved ultrasonic amplitudes (peak–peak) from virtual cross-sections. Note the difference in the shape of the curve depending on the position of the cross-section.

The characterization of the microstructured surface is based especially on geometrical aspects and on defined areal surface parameters. To analyze those values in detail, a laser scanning microscopy (LSM) image was taken with a Keyence VK-9700 (Keyence Corporation, Osaka, Japan) instrument applying a 50× objective in the middle of the milling path. At this position, the microstructure should be very close to the geometrical design due to the negligible effect of the path curvature. It should be noted that recutting by the tool was avoided, because its diameter was larger than the length of the specimen surface and the UVSM process was stopped directly above the middle of the surface. The size of the analyzed measurement field was reduced to 0.2 mm × 0.2 mm, since a sufficient number of relevant structural elements is thus represented in each direction. This image also allowed for cross-sections in the cutting direction, whereby the measured amplitude *A*_US_peak-peak_, which is defined in the case considered as the peak-to-peak value of the analyzed sinusoidal surface shape, could be determined. Based on five individual measurements, a mean value was calculated. However, it should be noted that measurements were taken at suitable locations: while values of around 4 µm were measured directly in the valley ground due to the smoothing effects caused by the cutting edge rounding (section A-A), the desired 5 µm can be detected close to the peaks of a path (section B-B), as shown in Figure 1. In addition, the different curve types can be seen, and some surface imperfections such as increased burr formation are marked as examples.

The following areal surface parameters according to DIN EN ISO 25178-2 [23] were taken into account, since they provide important information, especially considering the surface texture and shape of microstructures. Furthermore, they can possibly be associated with the coating adhesion behavior [24]:

- Maximum height *Sz* (simulation)/ten-point height *S10z* (measured surfaces);
- Developed interfacial area ratio *Sdr*;
- Texture aspect ratio *Str*;
- Root mean square gradient *Sdq*;
- Arithmetic mean peak curvature *Spc*.

Irregular surface deviations such as burrs can be detected using SEM micrographs or quantified using LSM images. Moreover, the reference surface section underwent an examination to determine the existing structural displacement. A “reference line” was utilized to measure this in units of length, which corresponds with the programming of the numerical simulation (Figure 1). As the size of the measuring section is significantly smaller in relation to the flight circle (=diameter) of the tool, the paths in the cutting direction can be regarded as almost straight or parallel, as the effect of the path curvature is irrelevant. This is taken up in the design of the FE model. A correlation of the material proportions was also conducted in addition to directly comparing the characteristic distribution.

#### 2.1.2. Tool Characterization

**Figure 2:**
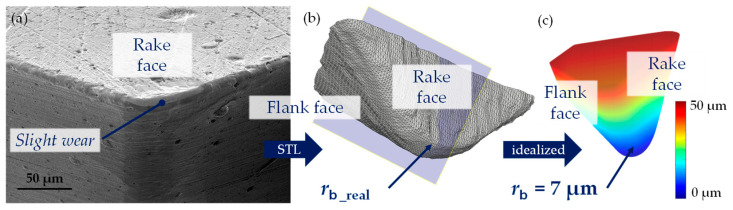
Tool geometry: an SEM micrograph of the tool used (**a**), the STL data set derived from LSM measurement (**b**) and the idealized tool model (**c**).

The tool used in the UVSM process for microstructuring the mentioned specimen was analyzed by SEM with an EVO MA25 (Carl Zeiss AG, Oberkochen, Germany) and LSM in unworn and worn conditions, Figure 2. Based on the geometrical specification of the uworn tool, especially the cutting edge radius, a virtual tool was designed. Therefore, the cutting edge radius was averaged from 20 profiles measured along/in the area of the corner radius and determined as *r*_b_ = 7 µm. The clearance angle was 40°, the rake angle 0° and both the angles of the major and minor cutting edges were 45°.

For the numerical simulation of the real surfaces, the tool used was imported as an STL file based on the 3D point cloud captured using LSM. As it is visible in the SEM micrograph (Figure 2a) and also in the cross-section of the LSM image (b), the cutting edge and the corner radius slightly change their shape toward the major cutting edge. This is due to the higher forces in the direction of feed motion. Additionally, the cutting edge radius increases or changes its form, respectively, and as a result the clearance angle decreases. This could lead to a smoothing effect especially in the area of the corner radius in the middle of a path in the cutting direction, if the aspect ratio of the microstructure (structural height/structural distance) is appropriate.

### 2.2. UVSM Trajectory and MATLAB-Based Kinematic Simulation

**Figure 3:**
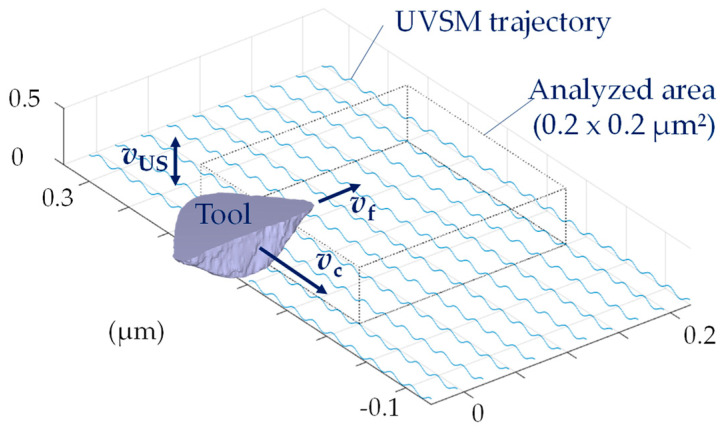
The schematic design of the UVSM trajectory and the analyzed area.

The kinematic simulation of the resulting microstructure with the software MATLAB (version R2023a, The MathWorks, Inc., Natick, MA, USA) is based on a Dexel model that was already described in [12], and it is shown schematically in Figure 3. The structural displacement and its influence on the characteristics of the microstructure have been analyzed in detail [13]. The structural displacement cannot be controlled for the UVSM in case of face milling, as, on the one hand, the spindle speed fluctuation of the real tool machine of ±3 min^−1^ has a significant influence, but, on the other hand, the frequency in the experimental part was a controlled process parameter and, therefore, it was variable. Thus, different values of the structural displacement must be taken into account in a comparative study. These are expressed by X.0, X.25 and X.5 for the kinematic simulation. The corresponding factor represents an integer multiple of the structural displacement in the cutting direction, which occurs in the next feed path.

### 2.3. FE Modeling and Methodology

#### 2.3.1. Model Geometry and Boundary Conditions

For developing the FE model, a numerical procedure was performed using the commercial software Abaqus (version 2020, Dassault Systemes, Johnston, RI, USA). As mentioned above, the 3D geometry of the tool was imported from the CAD software Autodesk Inventor (version 2018, Autodesk Inc., San Rafael, CA, USA), and two types of tool designs were considered: an ideal tool, based on geometrical information about the tool in unworn condition, and a tool exhibiting slight wear. The geometrical data were generated by LSM.

**Figure 4:**
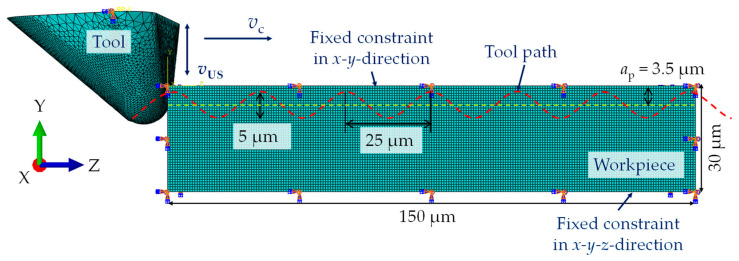
A schematic representation of FEM model: mesh, boundary conditions and tool path in cutting direction.

As shown in Figure 4, the tool moves in a sinusoidal path with an ultrasonic vibration amplitude of 5 µm (peak–peak). The frequency of 19.7 kHz and the cutting speed of *v*_c_ = 30 m/min led to structural distances of 25 µm. In order to study the microstructured surface after the machining process, a rectangular cuboid geometry with the dimensions 150 µm × 150 µm × 30 µm was considered as the workpiece. The tool was positioned so that the rake face was orthogonal to the cutting direction (Z-direction, Figure 4).

Within the numerical simulation model, an explicit dynamic analysis type was applied, which performs a large deformation with a large number of small time increments efficiently. Surface-to-surface interaction with the penalty contact method was applied considering a coefficient of friction of 0.4 [25]. The tool was defined as a rigid body. The outer surface of the whole workpiece was considered fixed in all directions of movement and rotation (U1 = U2 = U3 = UR1 = UR2 = UR3 = 0). The XY plane of the workpiece, where the tool comes into contact with the workpiece, was set to free in the Z-direction and fixed in the X- and Y-directions (U1 = U2 = UR1 = UR2 = UR3 = 0), as the tool needed to move in the Z-direction. As shown in the state of the art, one of the advantages of ultrasonic vibration superimposed machining is the reduction in temperature compared to conventional face milling. Therefore, no temperature effects such as heat input due to friction or plastic deformation were taken into account in this model. As a result, a constant temperature of 20 °C was defined within the simulation domains.

#### 2.3.2. Material Properties

The martensitic stainless steel (X46Cr13) was used as workpiece material. The indexable insert consisted of cemented carbide, coated with “TiXCo3”. The physical material properties, plasticity and damage data have a significant impact on the formation of the microstructure due to material removal and deformation, as shown by the simulation results. The relevant physical material properties are shown in Table 2. The material data for cemented carbide are listed for comparison with steel and because they are specified in Abaqus, even if they are not taken into account in the FE simulation when using the “rigid body” type for the tool.

The JC flow stress model was used to predict deformation at different strain rates for the plastic and damage behavior of the material. Since the material data for the JC plasticity and damage model of X46Cr13 stainless steel were unavailable, the values for 316L stainless steel were used instead, as 316L has comparable properties to X46Cr13. The JC equation for temperature-dependent plasticity is shown in Equation (1).

(1) $$\sigma =\left(A+B{\left(\varepsilon_{pl}\right)}^{n}\right)\left[1+Cln\left(\frac{\dot{\varepsilon_{pl}}}{{\dot{\varepsilon }}_{p0}}\right)\right]\left[1-{\left(\frac{\vartheta -\vartheta_{tr}}{\vartheta_{m}-\vartheta_{tr}}\right)}^{m}\right]$$

Within Equation (1), σ represents the flow stress in MPa, $\varepsilon_{pl}$ is the equivalent strain, $\dot{\varepsilon_{pl}}$ is the plastic strain rate in s^−1^ and ${\dot{\varepsilon }}_{p0}$ represents the effective plastic strain rate in s^−1^, which was used to determine the material parameters. ϑ (°C) is the running experiment temperature, and $\vartheta_{tr}$ (°C) is the transient temperature, which is defined as the temperature below which there is no temperature dependence on the expression of the flow stress. $\vartheta_{m}$ (°C) is the melting temperature of the material. *A*, *B*, *C*, *n* and *m* are the material constants of the workpiece [26]. The constants are listed in Table 3.

The JC damage model was used to perform the chip formation and separation. This model assumes that plastic strain is present at the onset of damage. The JC damage equation is shown in Equation (2).

(2) $$\varepsilon_{D}^{pl}=\left[D_{1}+D_{2}exp\left(D_{3}\frac{p}{q}\right)\right]\left[1+D_{4}ln\left(\frac{\dot{\varepsilon_{pl}}}{{\dot{\varepsilon }}_{p0}}\right)\right]\left[1-D_{5}{\left(\frac{\vartheta -\vartheta_{tr}}{\vartheta_{m}-\vartheta_{tr}}\right)}^{m}\right]$$

In Equation (2), *D*_1_–*D*_5_ are the failure parameters, p is the mean (or hydrostatic) stress and q is the von Mises stress. The parameters named in addition to the JC plasticity model are listed in Table 3.

#### 2.3.3. Simulation Procedure

For this type of simulation, an 8-node linear brick, reduced integration, hourglass control (C3D8R) mesh type is used for the workpiece, and a 4-node tetrahedron (C3D4) is utilized for the tool. A refined mesh with an element size of 0.7 µm is applied throughout the workpiece. The tool mesh is refined with an element size of 1 µm at the contact area with the workpiece and applied coarse at the non-contact area (refer to Figure 4) to save computing time.

**Figure 5:**
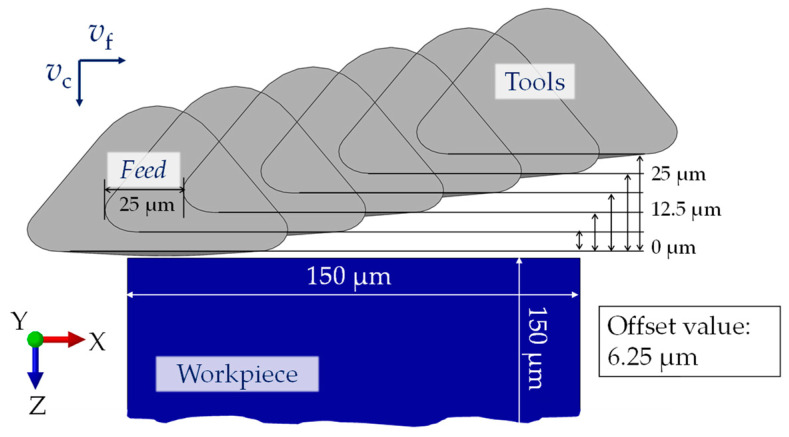
Arrangement of six tools with offset value of 6.25 µm (resulting structural displacement: X.75).

In the real UVSM process, the tool moves in three directions. Fluctuations in both rotational speed and ultrasonic frequency cause structural displacements from one feed path to the next feed path. To achieve this structural displacement in the simulations, the tools are arranged side by side in the direction of the feed motion with a distance (=offset value) equal to the wavelength of 25 µm (Figure 5). Furthermore, different offset values are set to achieve the desired structural displacements, listed in Table 4. Figure 5 illustrates the arrangement of six tools with a specified offset value of 6.25 µm relative to the workpiece. The use of multiple tools in simulations is necessary to replicate the structural displacement produced by a single tool moving as a feed in real experiments. By adjusting the spacing between the tools and their offset values, the desired structural displacement can be achieved.

For a numerical near-real simulation (nrS) with the real tool in worn condition, nine tools are considered, and their offset values, derived from experiments as can be seen in Figure 1, are also listed in Table 4. The relevant parameters employed in the simulation are summarized in Table 5. Based on this information, a direct comparison was conducted between the adjusted numerical simulation and the real surface section after machining.

While the depth of cut in the real UVSM process was 30 µm (the distance between the workpiece surface and the zero position of the oscillation), implementing such a depth in the simulation model would lead to computational challenges due to the severely increased number of elements required. To balance computational efficiency and accuracy, a depth of cut of *a*_p_ = 3.5 µm, which is 1 µm more as the ultrasonic amplitude *A*_US_, was employed throughout the numerical simulations. This ensured that the tool remained in contact with the workpiece and removed material, considering the opposing forces from the material during chip formation.

### 2.4. Postprocessing

**Figure 6:**
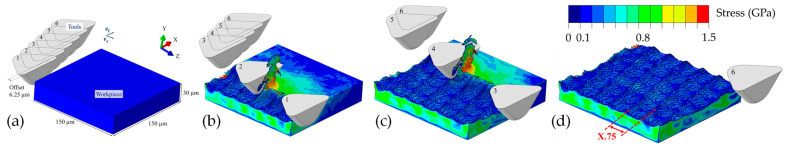
The FE simulation of the UVSM process for an offset value of 6.25 µm and a structural displacement of X.75, respectively, with an ideal tool: (**a**) the initial state of the tool and workpiece; (**b**) first tool exit and second tool entry; (**c**) third tool exit and fourth tool entry; (**d**) sixth tool exit forming the microstructure of the surface.

Figure 6 exemplarily shows a step-by-step evolution of the microstructure formation during the simulated UVSM process, incorporating the plastic deformation of the chip. The false color scale is a representation of the stress in GPa. In Figure 6a, the initial arrangement of the tools and the workpiece with an offset value of 0.25 µm, which provides a structural displacement of X.75, is exemplarily shown. This is followed by Figure 6b, where the first tool exits after cutting material from the workpiece. Simultaneously, the entry of the second tool is shown, accompanied by the cutting and deformation of the workpiece material to form an additional microstructure. The analysis of the von Mises stress shows that the greatest stress on the workpiece material prevails in the cutting zone as well as on the removing chip. Figure 6c again shows another state during processing by tool number four. The final state of the workpiece upon the sixth tool’s exit with a completely formed microstructure can be seen in Figure 6d.

**Figure 7:**
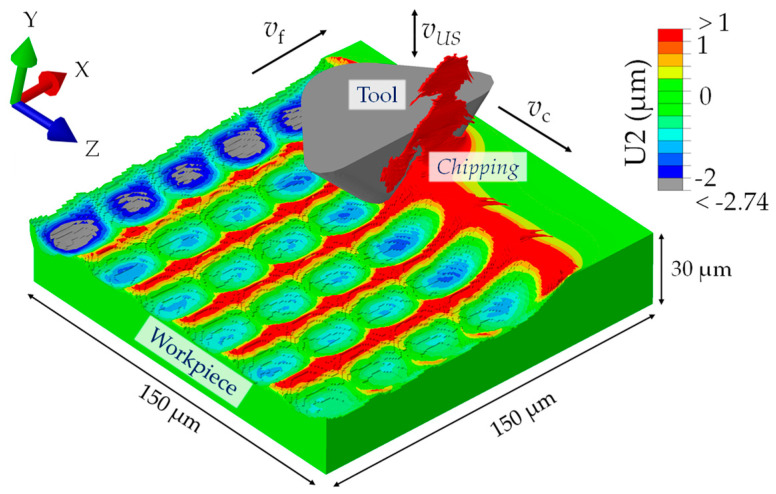
The FE simulation of the UVSM process with a structural displacement of X.0 and the “fifth tool” in motion. The displacement of the network elements from their original position in the Y-direction (U2) is represented.

Figure 7 shows the resulting microstructure formation for a structural displacement of X.0. The coloring of the surface represents the displacement of the network elements from their original position in the Y-direction (U2). It can be seen that the greatest displacements occur in particular at the structural peaks (red) and at the chip run-off, while the mesh elements in the valleys are pushed in a negative Y-direction.

To reduce the influence of boundary conditions on the extracted surface characteristics such as the profile height, only the central region of the workpiece with completely formed microstructures, excluding the edges, was considered for analysis. Impairing artifacts, such as chips, were removed for this purpose. As the element size is 0.7 µm for the workpiece, the nodal distance amounts to 0.7 µm. The nodal points of the microstructure surface of the workpiece were exported as cloud points for further analysis.

For filtering and characterization, the software MountainsMap^®^ (version 7.4, Digitial Surf, Besançon, France) was used. A uniform evaluation of the surface data was necessary, on the one hand, to increase the size of the section under consideration and, on the other hand, to set a uniform resolution. For this purpose, the operator “Stitching” was first used, whereby an automated composition was carried out on the basis of characteristic features of duplicated individual sections. Subsequently, the resolution was set to an X/Y distance of 1 µm by means of “Resampling”. This meant that the same data-related conditions prevailed for all the surfaces under consideration. After extracting a square area with a side the length mentioned (200 µm), the data were filtered by a robust Gaussian filter (3 µm) to remove the sub-roughness.

## 3. Results

### 3.1. Comparison of Areal Surface Parameters

**Figure 8:**
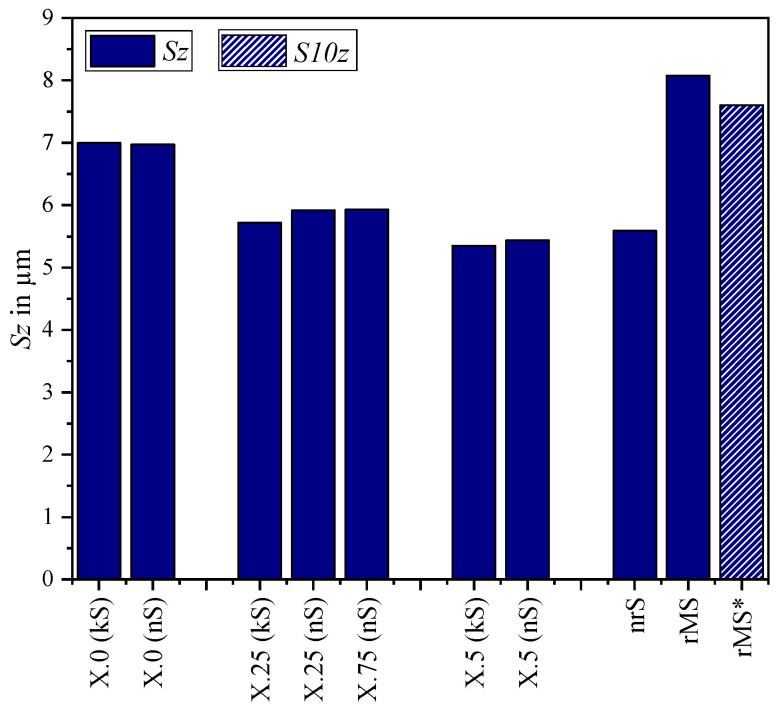
Profile height (*Sz* for simulations, * *S10z* for real surface) for different structural displacements.

**Figure 9:**
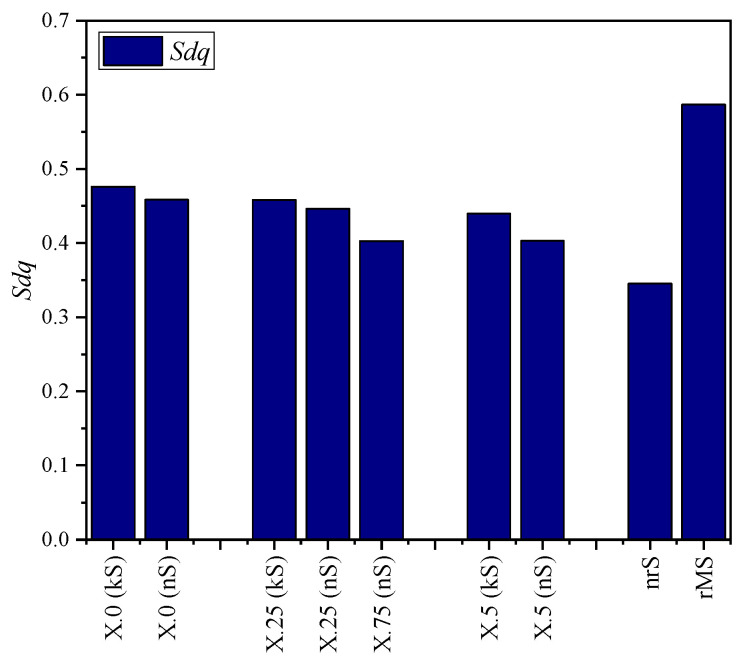
Root mean square gradient *Sdq* for different structural displacements.

**Figure 10:**
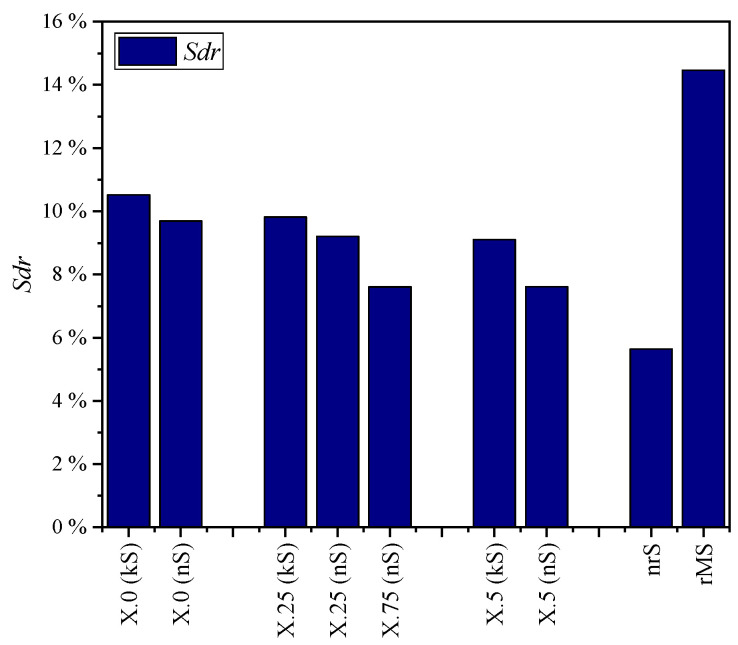
Developed interfacial area ratio *Sdr* for different structural displacements.

**Figure 11:**
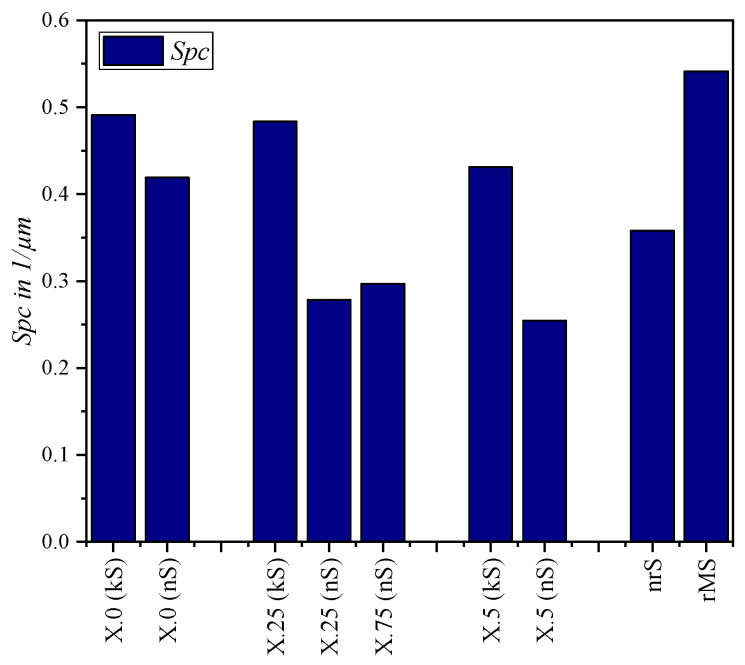
Arithmetic mean peak curvature *Spc* for different structural displacements.

**Figure 12:**
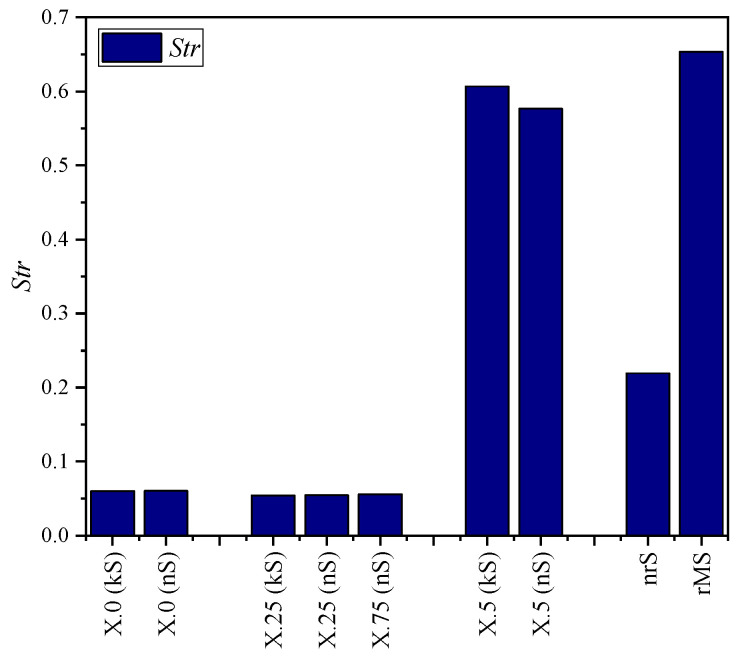
Texture aspect ratio *Str* for different structural displacements.

The quantitative evaluation of the kinematic (kS) and numerical simulated (nS) surfaces and the real microstructure (rMS) was carried out using the parameters presented in Section 2.1. These are compared in diagrams in Figure 8, Figure 9, Figure 10, Figure 11 and Figure 12.

When analyzing the maximum height *Sz*, the 10-point height *S10z* for the real surface was also considered, as this value is more robust against outliers. However, these values are not useful for simulated surfaces as there are no such outliers. It is clear that the values between kinematic and numerical simulation are in a similar range, but there are differences between the structural displacements. While the highest values occur with X.0, those of X.5 are the smallest. This can be explained by the summation of kinematic roughness and vibration amplitude and was already analyzed in [13]. There is a more distinct difference between the near-real numerical simulation and the real surfaces. While the microstructure simulated using Abaqus can be compared with the structural displacement of X.5 in terms of maximum height, the peaks of the real surface have the largest value. This can be explained by imperfections or irregularities such as burr formation, as can be seen in Figure 1.

The diagrams of the root mean square gradient *Sdq* (Figure 9) and the surface magnification *Sdr* (Figure 10) show almost analogous trends. The larger the structural displacement becomes, the greater are the differences in the flank steepness between the simulation methods and consequently also in the surface magnification.

The parameter *Spc* indicates more significant differences between the two simulation methods, Figure 11. A direct comparison shows that higher values for the arithmetic mean peak curvature are achieved with the purely kinematic approach, which means “sharper” peaks. This can be explained by the modeling method used in the numerical simulation. The existing element mesh is not only “separated”, but also undergoes a high proportion of “deformation”, whereby the mesh elements are distorted. This results in smoothing effects, which are expressed by this parameter. The higher *Spc* value of the real surface compared to the near-real simulation indicates a higher burr formation, whereby the peaks have smaller radii. This finding also correlates with the other surface parameters considered so far.

The texture analysis on the existence of a preferred direction again shows hardly any difference between the simulation approaches. It thus reflects existing findings on the influence of the structural displacements on the isotropy and emphasizes that its influence on the directional dependence of the microstructures is significantly greater than the consideration of deformative aspects. As expected, the microstructures with X.5 have almost no directional dependence, while the others are characterized by strong anisotropy. The striking thing about this assessment is, however, the comparison between real surfaces and near-real simulation. Here, the *Str* values significantly differ despite the same structural displacement. Once again, one possible explanation could be the insufficiently simulated burr formation and its irregularity in reality, which results in a considerably more isotropic microstructure.

### 3.2. Comparison of Material Distribution

**Figure 13:**
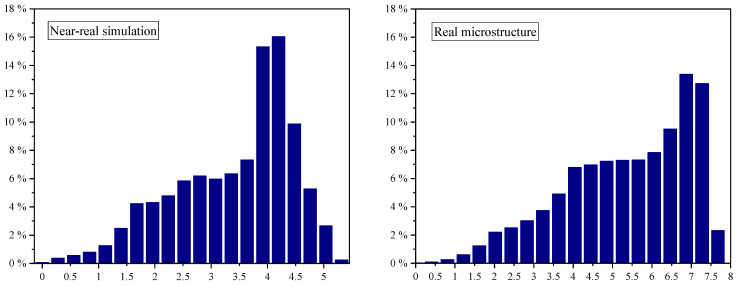
A comparison of the material distribution in the form of bar charts of the near-real simulation (**left**) and real microstructure (**right**).

In order to obtain a further statement on the comparability between the real microstructure and the numerical near-real simulated surface section, the material distribution was compared (Figure 13). This was also created using the MountainsMap^®^ software with the operator “Histogram”. This was applied to observe the density of the distribution of data points on the surface under investigation. The horizontal axis is shown in depth, while the vertical axis is shown as a percentage of the total height. The number of bars selected was 20 to achieve a representable ratio between a meaningful summary and depth of detail.

Although the direct height is different, as already determined in the analysis of the *Sz* or *S10z* values, respectively, the qualitative shape of the two curves shows a high level of agreement. This is also clear from the correlation coefficient according to Pearson. This was determined by the calculation of the correlation of the distributions, regardless of their height values. The resulting value of *r* = 0.6 indicates a moderately strong positive correlation between the two material distributions. It can be concluded from this that although the numerical near-real simulation shows deficits compared to reality in texture aspects and specific characteristics such as peak curvature and flank steepness, it basically produces a similar structural characteristic.

### 3.3. Qualitive Comparison

All simulated microstructures and their real surfaces are shown in Table 6. For visual comparability, the height scale, illustrated in (i), was normalized for all surfaces (from −10 µm to 10 µm) and the zero level was centered.

The visual similarities dominate in almost all simulated variants. Only a slightly greater sub-roughness is visually noticeable in the simulated surfaces with the FE model, which is mainly due to the simulation methodology by shifting the mesh elements.

**Figure 14:**
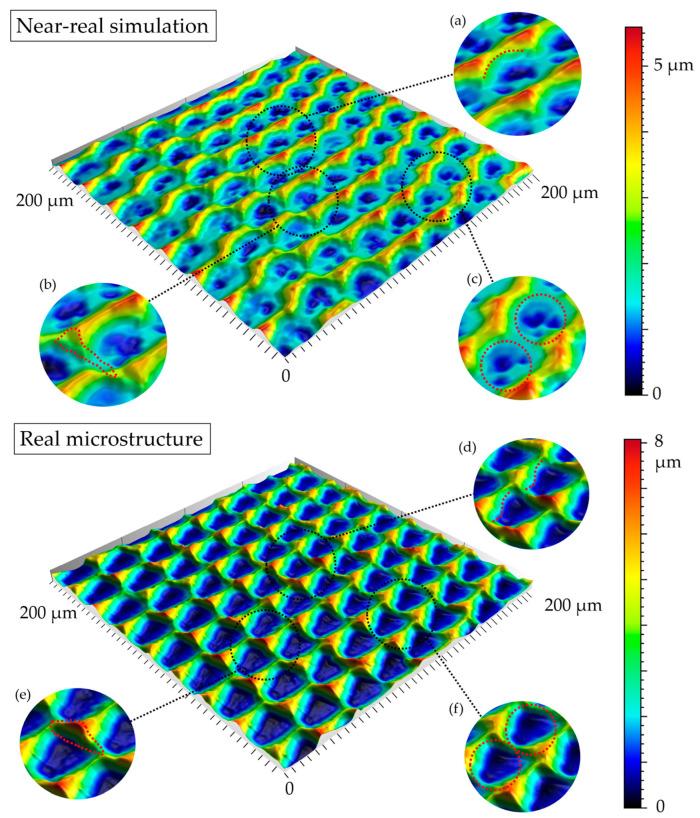
A visual comparison of the numerical near-real simulation and the real microstructure with regard to different details resulting from plastic deformation: impression of the tool corner on the flank in cutting direction (**a**, **d**); steepness of the flanks against the direction of feed motion (**b**, **e**); shape of the valleys (**c**, **f**).

However, a comparison of the near-real simulation with the real microstructure reveals some aspects that can explain the differences in the areal surface parameters. For this reason, both are shown again with their individual height scales, Figure 14. In particular, the following differences within the simulated microstructure are highlighted: (a) the “symmetrical representation” of the tool corner, (b) the less steep flanks against the direction of feed motion and (c) the “rugged” shape of the valleys.

All three characteristics, which are different in themselves, indicate that the influence of the plasticity in terms of shaping of the microstructure assumed in the FE model is lower than in the real material. The fact that the flanks of the real microstructure are significantly steeper (e), especially against the direction of feed motion, could be explained by a more pronounced plastic deformability of the real material. This also results in the more asymmetrical “impressions” of the tool corner in the real microstructured surface (d) in comparison to the simulation (a), as the shape is subsequently deformed to a greater extent. The shape of the valleys (c), which appears much more faceted in the simulation, although the geometric tool condition should be almost identical, also suggests that material separation occurs earlier. In reality, it is more likely that plastic deformation and therefore smoothing take place. The deviating depth of cut in the numerical FE model could have an additional influence: since the tool engages deeper in the material in reality, deformation can take place at a higher volume, and thus, this leads to increased burr formation.

## 4. Discussion and Conclusions

For a precise prediction of the resulting surface microstructure of an ultrasonic vibration superimposed machining (UVSM) process, an FE simulation model was developed. The model was based on the process kinematics, real surface information and material data from the literature. For the simulative works, a tool in the unworn condition and a tool with wear were integrated. The microstructures generated by FE simulation were analyzed in MountainsMap^®^ software and compared to kinematically simulated and real microstructures.

Apart from small differences in the characteristic values *Spc* and even smaller deviations for *Sdr*, which can be explained primarily by digital boundary conditions, there are no significant differences with regard to the investigated areal surface parameters. Although the two simulation methods are based on different assumptions (pure material separation model vs. elastic–plastic deformation processes), the resulting microstructures differ only slightly. While the plastic deformation and burr formation of the numerical simulation are visually recognizable, it has hardly any influence on the level of the characteristic values. In addition, the near-real simulation still exhibits considerable quantitative differences to the comparative real surface section, although this was oriented as closely as possible to reality according to the current state of knowledge. This deviation from reality clearly demonstrates that the purely kinematic aspects still have an excessive influence on the structural characteristics. This can be explained by the following causes:

- Material inhomogeneities in the machined material result in irregular burr formation, which cannot be represented in the FE model.
- The Johnson–Cook model describes the yield stress as a function of the strain rate and temperature. The defined model parameters (see Table 3) were assumed on the basis of literature research. However, it is possible that these parameters do not match the material behavior of the workpiece precisely enough. It should also be noted that the conditions under which the material parameters are determined are considered as a further problem or limit, respectively, as errors could occur here due to deviations between test conditions and real conditions.
- Temperature influences were not taken into account in the current analysis, so possible influences such as material softening could not be considered.
- The discrepancy between the real depth of cut and that used in the model is comparatively high. This circumstance may have a greater influence, especially on the burr formation, than originally assumed.
- Progressive tool wear is not taken into account.

The influence of the (changed) tool geometry decreases as a result of tool wear, mainly expressed by the effective clearance angle, as the clearance angle required to avoid a smoothing of the resulting microstructure in the cutting direction decreases. Furthermore, other machining parameters such as cutting speed and feed are usually associated with changed structural distances, which also have a significant influence on the deformation processes.

Moreover, it should be noted that the investigations and consequently the findings obtained have so far only been examined for this single microstructure variant. It can be assumed that the proportion of the influence of plastic deformation decreases as soon as, for example, the aspect ratio of the structures (height/distance) becomes smaller. One reason for this is that there are fewer peaks per surface section under consideration, where the main burr formation occurs. Further investigations can therefore follow on from this.

Summarized, it can be stated, on the one hand, that the deviation of the surface parameters between the two simulation methods is relatively small and the deviation of the simulation from reality is significantly greater. This is, among other things, attributed to the simplification of the simulation models. On the other hand, however, the suitability of the selected areal surface parameters for characterizing geometrically defined microstructures of this size and for detecting differences can certainly be questioned.

Regarding computing time, the FE approach typically requires significantly more effort (a range of several hours to a few days) than the MATLAB-based kinematic simulation (minute-range). Although the simulation results presented can still be regarded as capable of improvement, the model developed can already make an important contribution to the prediction of microstructures resulting from UVSM processing, since the surfaces simulated using FE come closer to reality than those simulated kinematically. On the contrary, various modifications, such as the use of other materials or models, other tool types or modified production parameters, as well as the consideration of temperature development, are possible with the acceptance of higher computing times.

In order to reduce the gap to reality, the above-mentioned aspects should be taken into account. Furthermore, the implementation of a function that sets a randomized offset value, for example, could increase predictability.

## Figures and Tables

**Table 1:** Representative microstructures from the literature.

Author	Process	Representative Microstructure
Zhu et al. [9]	Combined rotary spatial vibrations of the tool and servo motions of the workpiece	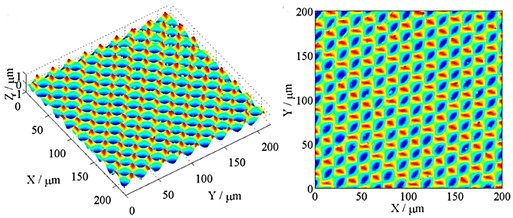
Yuan et al. [8]	2D compliant vibration-assisted cutting	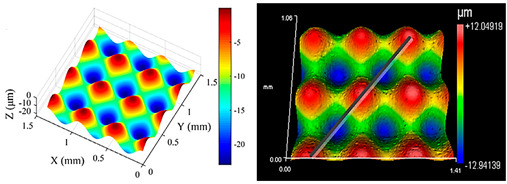
Liu et al. [10]	Radial ultrasonic vibration-assisted turning	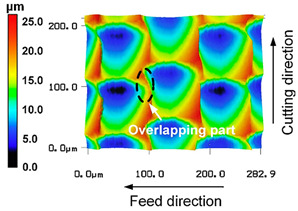
Zheng et al. [11]	Oblique rotary ultrasonic milling	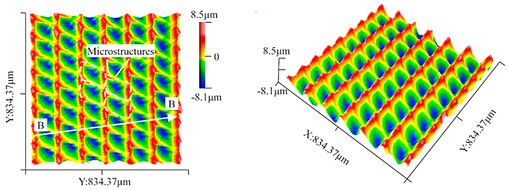

**Table 2:** The physical material properties of the steel X46Cr13 and the tool’s cutting material.

	Density (g/cm^3^)	Young’s Modulus (GPa)	Poisson’s Ratio
X46Cr13 [14]	7.8	200	0.3
Cemented carbide tool [26]	15	620	0.22

**Table 4:** Combinations of tools and displacements for FE model.

Parameter	Offset Value (µm)	Structural Displacement
Ideal tool (6 tools)	0	X.0
	18.75	X.25
	12.5	X.5
	6.25	X.75
Real tool (9 tools)	0, −9, −1, −19, −19, −20, −2, −12, −16	Alternating

**Table 5:** Relevant parameters used for FE model.

Parameter	Value
Cutting speed *v*_c_	30 m/min
Ultrasonic frequency *f*_US_	19.7 kHz
Amplitude *A*_US_	2.5 µm
Depth of cut *a*_p_	3.5 µm
Structural distance in the direction of feed motion	25 µm
Coefficient of friction	0.4

**Table 3:** Johnson–Cook plasticity parameters for 316L-grade stainless steel [26].

*A* (MPa)	*B* (MPa)	*C*	*n*	*m*	$\vartheta_{tr}$ (°C)	$\vartheta_{m}$ (°C)	${\dot{\varepsilon }}_{p0}$ (s^−1^)	*D*_1_	*D*_2_	*D*_3_	*D*_4_	*D*_5_
490	600	0.015	0.21	0.6	27	1454	1	0.05	3.44	2.12	0.002	0.61

**Table 6:** Visual comparison of all simulated surfaces and real microstructures with normalized height scaling; each surface section is 200 µm × 200 µm.

**(a) X.0 (kS)**	**(b) X.25 (kS)**	**(c) X.5 (kS)**
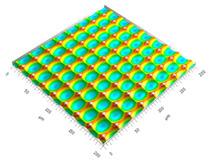	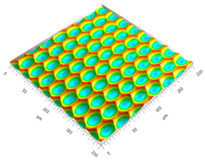	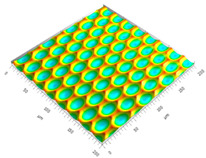
**(d) X.0 (nS)**	**(e) X.25 (nS)**	**(f) X.5 (nS)**
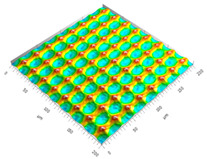	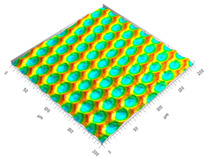	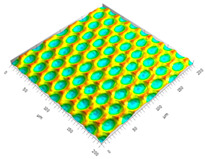
**(g) X.75 (nS)**	**(h) near-real simulation (nrS)**	**(i) real microstructure (rMS)**
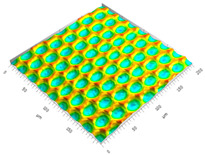	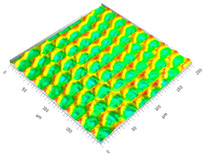	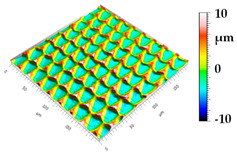

## Data Availability

The original contributions presented in the study are included in the article, further inquiries can be directed to the corresponding authors.

## Abbreviations

The following abbreviations and symbols are used in this manuscript:

**Abbreviation/Symbol**	**Definition**	**Unit**
*a*_p_	Depth of cut	µm
*A*_US_	Ultrasonic amplitude	µm
*A*_US_peak-peak_	Measured ultrasonic amplitude (peak–peak)	µm
$\dot{\varepsilon_{p0}}$	Effective plastic strain rate	s^−1^
$\varepsilon_{pl}$	Equivalent strain	-
$\dot{\varepsilon_{pl}}$	Plastic strain rate	s^−1^
*f*	Feed	µm
*f*us	Ultrasonic frequency	kHz
FE	Finite element	
JC	Johnson–Cook	
kS	Kinematic simulation	
LSM	Laser scanning microscopy	
nrS	Near-real simulation	
nS	Numerical simulation	
p	Mean or hydrostatic stress	MPa
q	von Mises stress	MPa
*r*	Correlation coefficient	-
rMS	Real microstructure	
*r*_b_	Cutting edge radius	µm
SEM	Scanning electron microscopy	
STL	Standard Transformation Language	
σ	Flow stress	MPa
ϑ	Running temperature	°C
ϑ_melt_	Melting temperature	°C
ϑ_tr_	Transient temperature	°C
UVSM	Ultrasonic vibration superimposed machining	
*v*_c_	Cutting speed	m/min
*v*_f_	Feed velocity	mm/min
*v*_US_	Velocity of the ultrasonic oscillation	m/min
		
*A*, *B*, *c*, *n* and *m*	Johnson–Cook plasticity material constants	
*D*_1_–*D*_5_	Johnson–Cook damage failure parameters	
*S10z*, *Sdr*, *Sdq*, *Spc*, *Str*, *Sz*	Areal surface parameters according to DIN EN ISO 25178-2	
U1, U2 and U3	Direction about the *x*-axis, *y*-axis and *z*-axis	
UR1, UR2 and UR3	Rotation about the *x*-axis, *y*-axis and *z*-axis	
